# Supplementary material for: Widespread premature transcription termination of Arabidopsis thaliana NLR genes by the spen protein FPA
Source: eLife. 2021 Apr 27;10:e65537. doi: 10.7554/eLife.65537 (PMC8116057; doi:10.7554/eLife.65537)
Supplement: Transparent reporting form [file elife-65537-transrepform.pdf]

eLIFE
1st Floor
24 Hills Road
Cambridge CB2 1JP, UK
P 01223 855340
W elifesciences.org
T @elife

eLife's transparent reporting form

We encourage authors to provide detailed information *within their submission* to facilitate the interpretation and replication of experiments. Authors can upload supporting documentation to indicate the use of appropriate reporting guidelines for health-related research (see EQUATOR Network), life science research (see the BioSharing Information Resource), or the ARRIVE guidelines for reporting work involving animal research. Where applicable, authors should refer to any relevant reporting standards documents in this form.

If you have any questions, please consult our Journal Policies and/or contact us: editorial@elifesciences.org.

Sample-size estimation

- You should state whether an appropriate sample size was computed when the study was being designed
- You should state the statistical method of sample size computation and any required assumptions
- If no explicit power analysis was used, you should describe how you decided what sample (replicate) size (number) to use

Please outline where this information can be found within the submission (e.g., sections or figure legends), or explain why this information doesn't apply to your submission:

No explicit power analysis was used. For the sequencing experiments, we had previously analyzed the properties of Illumina RNA-Seq data (Schurch et al., [2016] How many biological replicates are needed in an RNA-seq experiment and which differential expression tool should you use? ***RNA* 22**: 839-51). Consequently, we followed the recommendations of this study and used 6 biological replicates for each RNASeq condition here. No equivalent analysis of Helicos or nanopore DRS has yet been performed. In our previous studies on Helicos and nanopore DRS, we used 3 and 4 biological replicates, respectively, and were able to detect changes in 3' end position (Parker et al., [2020] Nanopore direct RNA sequencing maps the complexity of Arabidopsis mRNA processing and m6A modification. *eLife* 2020;9:e49658). We therefore used 3 or 4 biological replicates for Helicos and Nanopore DRS, respectively, in this manuscript, although replicates were pooled for statistical testing of 3' end profiles. Since our analysis of Pol II and FPA occupancy was qualitative, and the Pol II occupancy on Arabidopsis genes is well characterized, one replicate was used for ChIP-seq. We used four biological replicates for the LC-MS/MS analysis of m^6A according to our previous work and to previous analyses that established this approach (Parker et al., [2020] Nanopore direct RNA sequencing maps the complexity of Arabidopsis mRNA processing and m6A modification. *eLife* 2020;9:e49658; Huang et al., [2018] Recognition of RNA N6-methyladenosine by IGF2BP proteins enhances mRNA stability and translation. *Nat Cell Biol* 20, 285–295). For pathogen susceptibility experiments, the experiment was replicated three times, on each occasion the number of viable plants examined per genotype ranged from 7-45.

Replicates

- You should report how often each experiment was performed
- You should include a definition of biological versus technical replication
- The data obtained should be provided and sufficient information should be provided to indicate the number of independent biological and/or technical replicates

eLife Sciences Publications, Ltd is a limited liability non-profit non-stock corporation incorporated in the State of Delaware, USA, with company number 5030732, and is registered in the UK with company number FC030576 and branch number BR015634 at the address 1st Floor, 24 Hills Road, Cambridge CB2 1JP | August 2014

- If you encountered any outliers, you should describe how these were handled
- Criteria for exclusion/inclusion of data should be clearly stated
- High-throughput sequence data should be uploaded before submission, with a private link for reviewers provided (these are available from both GEO and ArrayExpress)

Please outline where this information can be found within the submission (e.g., sections or figure legends), or explain why this information doesn't apply to your submission:

Biological replicates for *IVI-MS*, Illumina RNA-Seq, Helicos DRS, nanopore DRS, m^6A LC-MS/MS and pathogen susceptibility experiments are stated in the main text, figure legends, and/or Materials and methods section. All sequencing statistics are also stated in Table S2. Technical replicates were not conducted. No outliers were removed.

Sequencing data has been made available through ENA and the accession number is stated in the Data Availability section. Due to the current inability to select the sequencing platform Helicos in the ENA upload metadata, the upload of Helicos data has been delayed. We therefore have made the data available on Zenodo ahead of ENA submission. Proteomics data has been made available on PRIDE, and the identifier is stated in the Data Availability section.

Statistical reporting

- Statistical analysis methods should be described and justified
- Raw data should be presented in figures whenever informative to do so (typically when N per group is less than 10)
- For each experiment, you should identify the statistical tests used, exact values of N, definitions of center, methods of multiple test correction, and dispersion and precision measures (e.g., mean, median, SD, SEM, confidence intervals; and, for the major substantive results, a measure of effect size (e.g., Pearson's r, Cohen's d)
- Report exact p-values wherever possible alongside the summary statistics and 95% confidence intervals. These should be reported for all key questions and not only when the p-value is less than 0.05.

Please outline where this information can be found within the submission (e.g., sections or figure legends), or explain why this information doesn't apply to your submission:

Detailed statistical analysis methods are outlined in Materials and methods.
Raw data is presented as overlayed stripcharts, or as a swarmplot, for boxplots/violinplots of single gene RNA-Seq and LC-MS/MS.
For pathogen susceptibility experiments, data is presented as a point plot showing median and 95% confidence intervals for each experimental replicate.
N values for histograms and metagene profiles are either labelled on figures or stated in the legend. Exact P values are reported in the main text.

(For large datasets, or papers with a very large number of statistical tests, you may upload a single table file with tests, Ns, etc., with reference to sections in the manuscript.)

Group allocation

- Indicate how samples were allocated into experimental groups (in the case of clinical studies, please specify allocation to treatment method); if randomization was used, please also state if restricted randomization was applied
- Indicate if masking was used during group allocation, data collection and/or data analysis

eLIFE
1st Floor
24 Hills Road
Cambridge CB2 1JP, UK
P 01223 855340
W elifesciences.org
T @elife

Please outline where this information can be found within the submission (e.g., sections or figure legends), or explain why this information doesn't apply to your submission:

Samples were assigned to groups based on genotype. No treatments were applied. Blinding was not used for data collection or analysis.

Additional data files ("source data")

- We encourage you to upload relevant additional data files, such as numerical data that are represented as a graph in a figure, or as a summary table
- Where provided, these should be in the most useful format, and they can be uploaded as "Source data" files linked to a main figure or table
- Include model definition files including the full list of parameters used
- Include code used for data analysis (e.g., R, MatLab)
- Avoid stating that data files are "available upon request"

Please indicate the figures or tables for which source data files have been provided:

All source code will be made available on GitHub as stated in the Materials and methods section.

Supplemental datasets for the following figures are provided:

Figure 1:

- Supplemental File 1 – FPA proteomics results

Figure 4:

- Figure 4 source data 1 – *fpa-8* vs Col-0 nanopore DRS 3' results,
- Figure 4 source data 2 – *35S::FPA:YFP* vs Col-0 nanopore DRS 3' results,
- Figure 4 source data 3 – *fpa-8* vs Col-0 Helicos DRS 3' results,
- Figure 4 source data 4 – *35S::FPA:YFP* vs Col-0 Helicos DRS 3' results,
- Figure 4 source data 5 – *fpa-8* vs Col-0 Illumina expressed region results,
- Figure 4 source data 6 – *35S::FPA:YFP* vs Col-0 Illumina expressed region results,
- Figure 4 source data 7 – *fpa-8* vs Col-0 Illumina splice junction results,
- Figure 4 source data 8 – *35S::FPA:YFP* vs Col-0 Illumina splice junction results.

Figure S4:

- Figure 4 source data 9 – Col-0, *fpa-8* and *35S::FPA:YFP* LC-MS/MS m^6A results

Figure 7:

- Figure 7 source data 1 – *ibm1-4* vs Col-0 $H3K9me^2$ results.

Figure 8:

- Figure 8 source data 1 – Col-0, Ksk-1, *fpa-7*, *fpa-8*, *pFPA::FPA* and *35S::FPA:YFP* *Hpa*-Hiks1 susceptibility results

eLife Sciences Publications, Ltd is a limited liability non-profit non-stock corporation incorporated in the State of Delaware, USA, with company number 5030732, and is registered in the UK with company number FC030576 and branch number BR015634 at the address 1st Floor, 24 Hills Road, Cambridge CB2 1JP | August 2014
